# Structure-Function Relationships in Temperature Effects on Bacterial Luciferases: Nothing Is Perfect

**DOI:** 10.3390/ijms23158119

**Published:** 2022-07-23

**Authors:** Anna A. Deeva, Albert E. Lisitsa, Lev A. Sukovatyi, Tatiana N. Melnik, Valentina A. Kratasyuk, Elena V. Nemtseva

**Affiliations:** 1 Biophysics Department, Siberian Federal University, 660041 Krasnoyarsk, Russia; alisitsa@sfu-kras.ru (A.E.L.); lsukovatyy@sfu-kras.ru (L.A.S.); valkrat@mail.ru (V.A.K.); enemtseva@sfu-kras.ru (E.V.N.); 2 Institute of Protein Research, Russian Academy of Sciences, 142290 Pushchino, Russia; tmelnik@vega.protres.ru; 3 Institute of Biophysics, Siberian Branch of Russian Academy of Sciences, 660036 Krasnoyarsk, Russia

**Keywords:** bacterial luciferase, enzyme activity, protein stability, sucrose, thermal inactivation, thermal denaturation, differential scanning calorimetry, molecular dynamics

## Abstract

The evaluation of temperature effects on the structure and function of enzymes is necessary to understand the mechanisms underlying their adaptation to a constantly changing environment. In the current study, we investigated the influence of temperature variation on the activity, structural dynamics, thermal inactivation and denaturation of *Photobacterium leiognathi* and *Vibrio harveyi* luciferases belonging to different subfamilies, as well as the role of sucrose in maintaining the enzymes functioning and stability. We used the stopped-flow technique, differential scanning calorimetry and molecular dynamics to study the activity, inactivation rate, denaturation and structural features of the enzymes under various temperatures. It was found that *P. leiognathi* luciferase resembles the properties of cold-adapted enzymes with high activity in a narrow temperature range and slightly lower thermal stability than *V. harveyi* luciferase, which is less active, but more thermostable. Differences in activity at the studied temperatures can be associated with the peculiarities of the mobile loop conformational changes. The presence of sucrose does not provide an advantage in activity but increases the stability of the enzymes. Differential scanning calorimetry experiments showed that luciferases probably follow different denaturation schemes.

## 1. Introduction

Living systems have highly sensitive regulatory mechanisms to perceive a wide range of environmental conditions and quickly adapt to even the slightest changes. In particular, microorganisms can tune the salt concentration, density, viscosity and other characteristics of the cell matrix in response to acute stress or during self-regulating processes that enable them to maintain homeostasis [1,2,3,4]. One of the most important parameters that affects microbial metabolism and proliferation is temperature. It is generally accepted to distinguish microorganisms into psychrophiles, mesophiles, and thermophiles according to the range of temperatures which they can inhabit. The accumulation of water-soluble organic compounds either by de novo synthesis or by consumption from the surrounding environment is a common mechanism of osmoregulation, UV-protection, and thermal adaptation among extremophiles and mesophiles as well (including *Bacteria*, some *Eukaryota* and a few methanogenic *Archaea* species) [5]. These compounds, so called extremolytes, are of different chemical structure, comprising saccharides, polyols, heterosides, amino acids, and their derivatives [6]. The adjustments occurring in the cell are aimed at maintaining various parameters including viscosity, which is crucial for diffusion-controlled reactions [3], as well as the structure and functions of cytosol components and mainly proteins [7,8]. Furthermore, proteins themselves could be adapted to temperature shifts that affect their folding, structure, dynamics and functional features [9]. The current work focuses on temperature effects on the properties of bacterial luciferases.

Luciferases are responsible for light emitting reactions in luminous organisms, among which bacteria are the most ubiquitous. There are more than 25 species of bioluminescent bacteria found within five genera: *Aliivibrio*, *Vibrio*, and *Photobacterium* within the *Vibrionaceae* family, *Photorhabdus* within the *Enterobacteriaceae* family, and *Shewanella* within the *Shewanellaceae* family [10]. They can be successfully isolated in different seasons and at various depths from the warm coastal and pelagic waters, as well as from the northern seas of the global ocean [11,12]. There are also freshwater and terrestrial luminous bacteria, and the latter group consists of only *Photorhabdus* species that are parasites infecting human skin lesions or insects with nematodes as the intermediate host [13]. Cold adapted representatives can be found in all known genera except the mentioned *Photorhabdus*. Recent studies also revealed several dozens of psychrophilic luminous strains, including first luminous *Kosakonia cowanii*, associated with the digestive system of fishes from the White, Okhotsk, and Bering Seas [14,15]. Thus, luminous bacteria have adapted to a wide range of temperatures, from near zero to about 35 °C, although thermophilic species have not been identified.

Bacterial luciferase is a heterodimeric enzyme that catalyzes the oxidation of reduced flavin mononucleotide (FMNH^−^), the monooxygenation of a long chain aliphatic aldehyde and the reduction of molecular oxygen resulting in the formation of the electronically excited intermediate that emits light [16]. The stability of luciferase to thermal inactivation in vitro could vary depending on the bacterial species from which it was isolated. For example, it was obtained that the remaining activity is higher for *Vibrio* (*Beneckea*) *harveyi* luciferase than for *Photobacterium phosphoreum* and *Aliivibrio* (*Photobacterium*) *fischeri* [17]. However, *V. harveyi* luciferase is less thermostable than its close homolog from *Vibrio campbellii* that differs by only eight amino acid residues (98.8% sequence identity) [18]. Also, *Photorhabdus luminescens* luciferase is remarkably more thermostable compared to *V. harveyi*, while their α-subunits share over 85% amino acid sequence identity [19]. A recent phylogenetic analysis of luciferase amino acid sequences showed that they fall into two highly conserved groups with a specific structure of the active site [20]. Each group comprises luciferases with the similar type of decay kinetics in reaction with dodecanal: “fast” type from the *Aliivibrio* and *Photobacterium* species, and “slow”—from *Vibrio* and *Photorhabdus* [21,22,23]. The majority of known psychrophilic luciferases belong to the group with the fast decay kinetics, however a few species with “slow” luciferase were also isolated from the northern latitudes, for example, *Vibrio splendidus* [15]. The presence of both types of luciferases among psychrophilic strains suggests that either luciferases acquired the properties of cold-active enzymes, regardless of the structural features of the active center, or the adaptation of luminous bacteria passed through a change in the other cellular components and did not affect the bioluminescent system.

This research aimed to reveal the effect of temperature on the reaction kinetics and structure of two types of bacterial luciferases and the role of extremolytes in maintaining the activity of these enzymes under unfavorable temperature conditions. We studied *Vibrio harveyi* luciferase characterized by the slow decay kinetics and *Photobacterium leiognathi* defined as the “fast” enzyme. Sucrose was chosen as a model extremolyte, since its buffer solutions proved to have no inhibitory effect on bacterial luciferase activity in contrast to glycerol solutions [24].

Our data showed that “fast” *P. leiognathi* luciferase remains active at relatively low temperatures, while the temperature range of “slow” *V. harveyi* luciferase functioning is shifted to higher temperatures. Molecular dynamics simulations revealed structural motifs, which could be critical for the activity maintenance at the corresponding temperatures: a mobile loop forming the active center and a hairpin loop located near the α/β-interface. We also found that the addition of sucrose significantly reduces the mobile loop fluctuations in the *P. leiognathi* structure at high temperature. On the contrary, sucrose was less effective in stabilizing both flexible motifs of *V. harveyi* luciferase. The analysis of thermal inactivation rate and denaturation thermodynamics of the luciferases revealed that *V. harveyi* enzyme is more thermostable. Sucrose was found to have a stabilizing effect against thermal inactivation and the denaturation of both luciferases. Thus, we carried out a detailed comparative study of the temperature effects on two types of bacterial luciferase that have never been performed before.

## 2. Results

### 2.1. Activity of V. harveyi and P. leiognathi Luciferases at Different Temperatures

**Figure 1:**
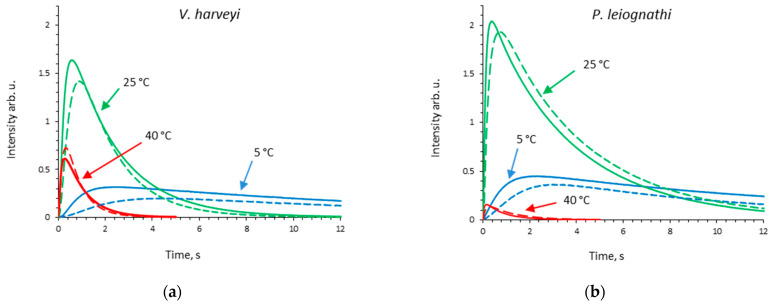
The kinetics of bioluminescence emission in reactions, catalyzed by luciferases from *V. harveyi* (**a**) and *P. leiognathi* (**b**) at low (5 °C, blue lines), medium (25 °C, green lines) and high (40 °C, red lines) temperature in the buffer (solid lines) and in sucrose solution (dashed lines). Concentrations in the reaction mixture were: luciferase—0.5 µM, flavin—15 µM, decanal—50 µM.

**Figure 2:**
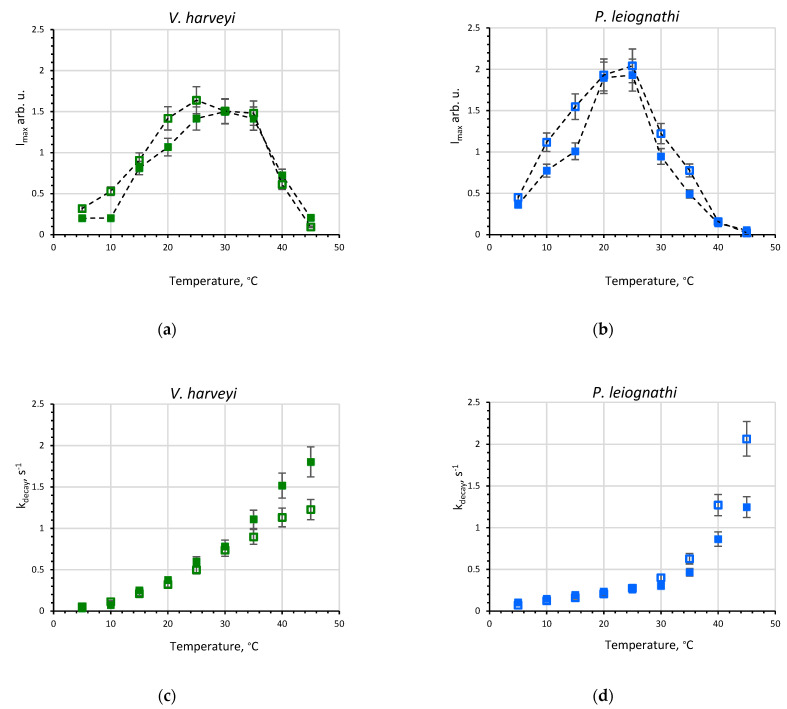
Dependence of kinetic parameters of luciferases from *V. harveyi* (**a**,**c**) and *P. leiognathi* (**b**,**d**) on temperature in the buffer (empty squares) and sucrose solution (filled squares): the peak intensity, I_max_, (**a**,**b**) and the decay constant, k_decay_, (**c**,**d**). The dashed lines are a guide for the eye. Concentrations in the reaction mixture were: luciferase—0.5 µM, flavin—15 µM, decanal—50 µM.

The kinetics of light emission in reactions catalyzed by *V. harveyi* or *P. leiognathi* luciferase was studied in the buffer and 30 (*w*/*w*)% sucrose at temperatures in the range of 5–45 °C. After mixing with substrates, bacterial luciferase can only make a single turnover due to the quick autoxidation of unbound FMNH^−^. Hence, the reaction kinetics is flash-like with a rapid increase succeeded by a relatively slow decay of light intensity (Figure 1). The following empirical kinetic parameters are estimated: the peak intensity (I_max_), the total quantum yield (Q*), and the decay constant (k_decay_). Their dependences on temperature are shown in Figure 2 and Figure S1 (in the Supplementary Material).

Comparing luciferases in the buffer, one can see that according to I_max_ *V. harveyi* luciferase demonstrates its highest level of activity over a wider temperature range than *P. leiognathi* enzyme (20–35 °C vs. 20–25 °C, Figure 2a,b). However, the amount of emitted light in a single turnover, Q*, in the range 10–25 °C is about twofold less for *V. harveyi* enzyme than for *P. leiognathi* one, but becomes almost equal in value for both luciferases at higher temperatures (Figure S1a,b). The decay rate, k_decay_, increases under higher temperatures (≥35 °C) more sharply for *P. leiognathi* luciferase (Figure 2d). Thus, in spite of high homology, *P. leiognathi* luciferase exhibits the properties of a more thermolabile enzyme than the *V. harveyi* one.

To mimic a viscous environment and the extremolyte effects on bioluminescent reactions from two bacterial species, we studied reaction kinetics in sucrose solution (Figure 1). A concentration of 30% was used, which corresponded to 0.99 M of sucrose and gave more than a threefold higher viscosity as compared with the buffer. From the kinetic curves in Figure 1, one can see that the reaction noticeably slows down in a more viscous medium for both luciferases, which is due to the diffusion control of some reaction steps [24]. The change of kinetic parameters in sucrose solution over the entire temperature range is shown in Figure 2 and Figure S1. We observed that in the presence of sucrose the peak intensity decreases at low temperatures (for *V. harveyi* luciferase—in the range 5–20 °C, for *P. leiognathi* one—in the range 5–15 °C) (Figure 2a,b), while the effect on the decay constant appears only at high temperatures (Figure 2c,d). All of this leads to a decrease of the reaction quantum yield in sucrose solution at lower temperatures for both luciferases (Figure S1). Interestingly, at temperatures >30 °C in the presence of sucrose, k_decay_ is higher for *V. harveyi* luciferase and lower for the *P. leiognathi* one, as compared with the buffer.

The observed temperature effects on the activity of the enzymes indicate that (i) *P. leiognathi* luciferase is more sensitive to temperature change than the *V. harveyi* enzyme and (ii) viscous sucrose solution gives no advantages for bacterial luciferases’ functioning either under “hot” (35–45 °C) or in “cold” (5–15 °C) conditions. These facts will be discussed in Section 3.

### 2.2. Thermal Inactivation of V. harveyi and P. leiognathi Luciferases

**Figure 3:**
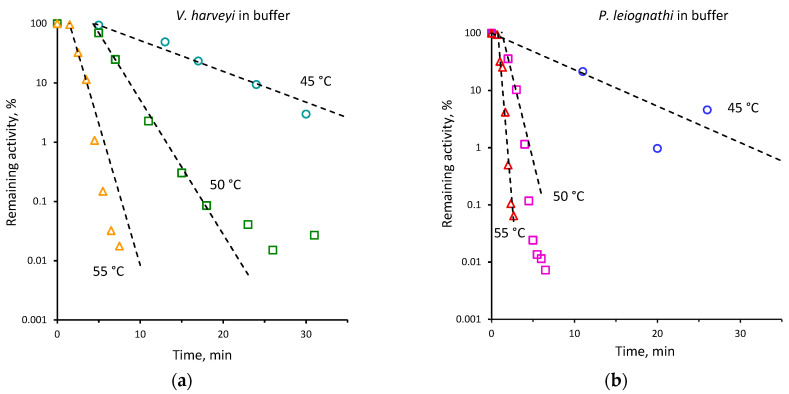

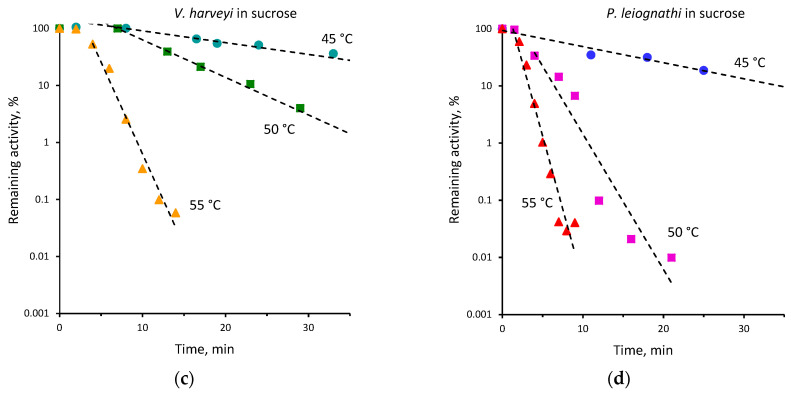
The dependence of the remaining activity of *V. harveyi* (**a**,**c**) and *P. leiognathi* (**b**,**d**) luciferases in the buffer (**a**,**b**, empty markers) and sucrose solution (**c**,**d**, filled markers) on the incubation time at selected temperatures (45 °C—circles, 50 °C—squares, 55°C—triangles) in semi-logarithmic scales. Lines refer to approximations with exponential function. Concentrations in the reaction mixture were: luciferase—0.5 µM, flavin—15 µM, decanal—50 µM. The activity was measured at 20 °C after the immediate cooling of the incubated enzyme.

We measured the remaining activity of *V. harveyi* and *P. leiognathi* luciferases at 20 °C after incubating the enzymes for different time intervals at various temperatures in the absence and presence of sucrose. The remaining activity at a certain time was expressed as percent of activity in the initial moment (i.e., without incubation), which is described in detail in Section 4.2. The time course of thermal inactivation of the enzymes at different temperatures is shown in Figure 3.

It is worth noting that for both enzymes we observed an induction phase (from 0.5 to 8 min, depending on temperature) (Figure 3). Incubation during this time resulted in the remaining activity of about 100%, indicating that the most thermally inactivated molecules recovered after the immediate cooling of incubated enzymes. The longer heat treatment resulted in a consistent decrease of the remaining activity, which was well described by a single exponential function. The corresponding rate constants of thermal inactivation of the bacterial luciferases are shown in Table 1. In a few cases an additional slow phase of inactivation was observed for both enzymes on further heating, namely at 50 °C (Figure 3a,c). Such an effect is usually explained by the aggregation of the protein molecules that prevents their refolding [25]. These data points were not included in the estimation of the inactivation rate constants as well as the points of the induction phase.

Comparing thermal inactivation of two luciferases, we found that the *V. harveyi* enzyme is less sensitive to heating, since its remaining activity was always greater than that of the *P. leiognathi* enzyme under the same conditions. The presence of 30% sucrose appeared to reduce the rate of thermal inactivation of both luciferases (Table 1 and Figure 3c,d). The effect was the most pronounced for *P. leiognathi* luciferase at 50 °C. In this case, enzyme inactivation slowed down more than four times.

**Figure 4:**
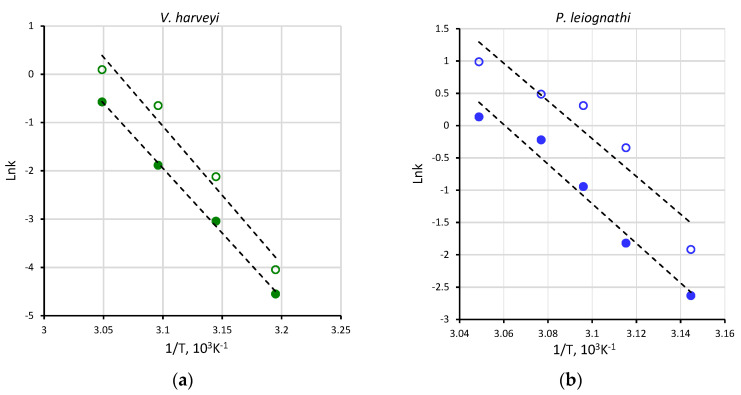
Arrhenius plot of the thermal inactivation of *V. harveyi* (**a**) and *P. leiognathi* (**b**) luciferases in the buffer (empty circles) and sucrose solution (filled circles). The k is the enzyme thermal inactivation rate constant at each respective temperature, taken from Table 1. Dashed lines refer to a linear approximation.

Using the rate constants, we performed the Arrhenius analysis of thermal inactivation of the two luciferases in the buffer and sucrose solution (Figure 4).

In spite of the protective effect of sucrose, evident from the decrease of the inactivation rate constants (Table 1), it was revealed that the activation energy of thermal inactivation, E_a_, does not change in viscous medium. In particular, E_a_ in the buffer and sucrose solution was found to be 237 ± 30 and 224 ± 7 kJ/mol for *V. harveyi* luciferase and 255 ± 27 and 243 ± 47 kJ/mol for *P. leiognathi* luciferase, respectively. Such estimations do not allow for the distinguishing of the degree of thermal stability of the two luciferases as well.

### 2.3. Thermal Stability of Luciferases as Revealed by Differential Scanning Calorimetry

**Figure 5:**
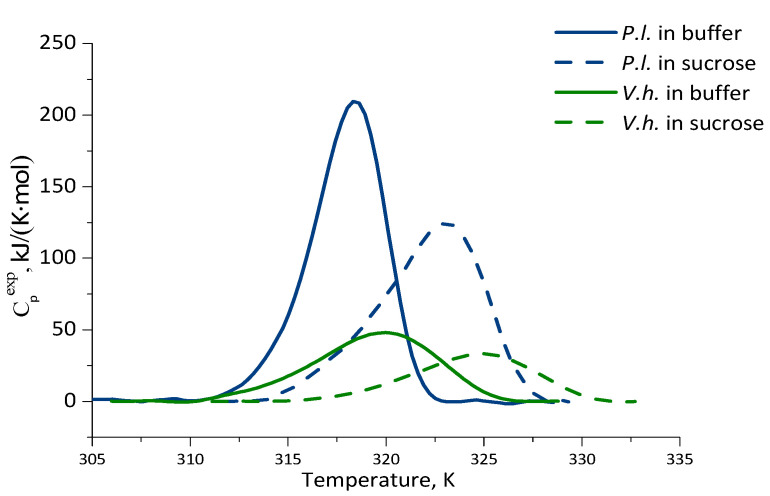
Temperature dependence of excess heat capacity functions for bacterial luciferases from *V. harveyi* (green lines) and *P. leiognathi* (blue lines) in the buffer (solid lines) and in 30% sucrose (dashed lines) at pH 6.9.

To compare thermal stability of the luciferases, their denaturation was studied by differential scanning calorimetry. Figure 5 shows the temperature dependencies of excess heat capacity functions (C_p_^exp^) obtained for bacterial luciferases at pH 6.9 in the buffer and in sucrose solution. It can be seen that the curves demonstrate peaks of cooperative heat absorption corresponding to protein denaturation. In Table 2 the thermodynamic parameters of the thermal denaturation of the luciferases are summarized. All samples are characterized by the lack of calorimetric reversibility and an asymmetry of the heat absorption peaks. The degree of peak asymmetry (ΔQ_−_/ΔQ_+_) is shown in Table 2. Generally, the asymmetry of the heat absorption peaks can be associated either with the non-equilibrium of the denaturation process or with the accumulation during denaturation of one or more intermediate states of different stability. Nevertheless, it can be concluded that *V. harveyi* luciferase has slightly higher temperature stability than the *P. leiognathi* one, both in buffer and in 30% sucrose. However, the enthalpy of thermal denaturation is greater in *P. leiognathi* luciferase than in *V. harveyi*. This indicates that the amount of the interactions (hydrophobic, van der Waals) that disrupt with increasing temperature is greater in *P. leiognathi* luciferase than in the *V. harveyi* one. The thermal denaturation of two luciferases also differs in the degree of cooperativeness: *P. leiognathi* luciferase has a higher cooperativeness (ΔT_1/2_ is less), i.e., the destruction of the native structure occurs in a narrower temperature range.

The addition of 30% sucrose to the buffer stabilized the luciferases: their T_m_ shifted by 4.7 K (*P. leiognathi*) and 5.0 K (*V. harveyi*). However, the sucrose effects on the process of thermal denaturation of two bacterial luciferases are different. In *P. leiognathi* enzyme, the addition of sucrose led to a broadening of the peak and an increase of its asymmetry. Such an effect of organic substances on the heat absorption curve is characteristic; it was observed on different proteins and explained by a slowdown of the denaturation kinetics process [26]. In the case of *V. harveyi* luciferase, the addition of 30% sucrose did not change either ΔT_1/2_ or ΔQ_−_/ΔQ_+_. Thus, our DSC experiments indicate that the protein denaturation schemes could be different for two studied luciferases.

### 2.4. Temperature Dependent Molecular Dynamics of V. harveyi and P. leiognathi Luciferases

To evaluate the role of structural dynamics of the proteins in experimentally obtained patterns, we conducted a molecular dynamics (MD) computation of two bacterial luciferases surrounded by water molecules at low (5, 15 °C), medium (27 °C) and high (45, 60 °C) temperatures.

Firstly, based on the obtained MD trajectories, we analyzed three parameters: the root-mean-square deviation of the backbone atoms (RMSD), the radius of gyration (R_g_), and the solvent accessible surface area (SASA). It allowed assessing the protein stability in the course of molecular dynamics and overall structure change. The results for the last 10 ns of the simulations are summarized in Table S1. No essential change of RMSD and R_g_ values for both enzymes was detected with increasing temperature. The RMSD values of *V. harveyi* luciferase were found to be lower than that of *P. leiognathi* luciferase (1.6–2.3 Å vs. 1.9–2.6 Å, respectively) and at 60 °C the standard deviation of the RMSD of *V. harveyi* luciferase appeared to be less than that of the *P. leiognathi* enzyme. It could reflect less deviation of the *V. harveyi* luciferase structure from the initial one at higher temperatures. The SASA values were obtained in the ranges of 284–290 and 293–301 × 10^2^ Å^2^ for the *V. harveyi* and *P. leiognathi* luciferases, respectively. It indicates that the last enzyme could be less compact.

Additionally, the molecular dynamics of the two luciferases surrounded by the mixture of water and sucrose molecules (corresponding to concentration 30%) was analyzed at 27 °C and 60 °C. It is worth noting that at high temperature the presence of sucrose reduced the deviation of SASA from the average value for *V. harveyi* luciferase (Table S1), while for the *P. leiognathi* enzyme there was no effect.

**Figure 6:**
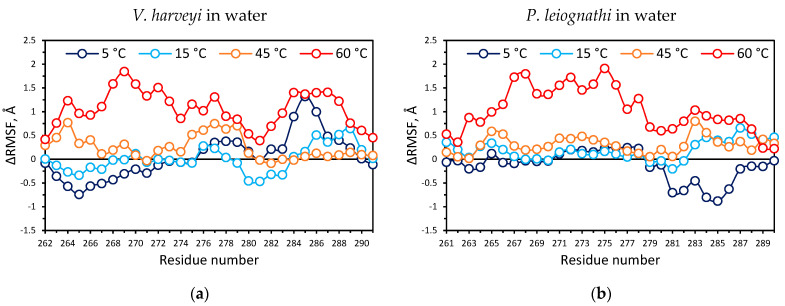

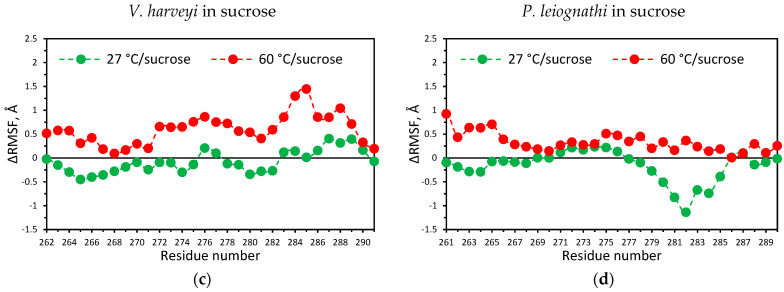
ΔRMSF of C_α_ atoms of *V. harveyi* luciferase mobile loop (262–291 a. r.) in water (**a**) and water-sucrose (**c**) and *P. leiognathi* luciferase mobile loop (261–290 a. r.) in water (**b**) and water-sucrose (**d**) at different temperatures. The positive value of ΔRMSF corresponds to a more flexible segment as compared with the structure in water at 27 °C, while the negative ΔRMSF corresponds to a more rigid segment. Correlation coefficients between the ΔRMSF profiles of *V. harveyi* and *P. leiognathi* mobile loops are presented in Table S2.

Secondly, we calculated the average root-mean-square fluctuations (RMSF) of C_α_ atoms of the luciferases in order to examine the structural flexibility of protein segments at different temperatures. For each temperature the change of flexibility was estimated as a difference according to ΔRMSF = RMSF_T_ − RMSF_27_, where RMSF_T_ and RMSF_27_ are parameters for temperature T and 27 °C, respectively. In Figures S2 and S3 for two luciferases ΔRMSF of an α-subunit, which bears the enzyme active center, and a β-subunit, which stabilizes active enzyme, are shown [27]. The temperature effects on flexibility of a functional mobile loop in α-subunits were found for both enzymes (Figure 6a,b). The increased fluctuation of the region near αHis150 in *V. harveyi* luciferase was also observed (Figure S2). This amino acid residue is located at the apex of the hairpin loop structure (142–160 a. r.), which passes along the periphery of an α/β-interface of the luciferase [28]. Significant fluctuations in this region at 45 °C could expose a part of the α/β-interface to the solvent molecules, which could affect the enzyme activity.

The mobile loop is a particularly important functional region adjacent to the active site of the luciferase. It undergoes conformational changes upon enzyme-substrate complex formation [29]. We observed that at 5 °C two segments of the mobile loop change their flexibility in different manners as compared to 27 °C. The loop segment about 260–275 a. r. became less mobile in *V. harveyi* luciferase (Figure 6a), while its rigidity in the *P. leiognathi* enzyme did not change (Figure 6b). The loop segment at about 275–290 a. r., on the contrary, had a higher mobility in *V. harveyi* luciferase (Figure 6a) and a lower mobility in *P. leiognathi* enzyme under low temperatures (Figure 6b). Hence, we revealed that the mobile loop flexibility differs between the two luciferases at 5 °C, and there is moderate negative correlation between the ΔRMSF profiles of *P. leiognathi* and *V. harveyi* mobile loop segments, with a correlation coefficient of −0.52 (Table S2).

Inspecting the possible influence of sucrose on the mobile loop of the luciferase, we compared ΔRMSF of the proteins surrounded by water and sucrose-water mixture at 27 and 60 °C (Figure 6c,d, Figures S4 and S5). The obtained data indicate that the flexibility of the mobile loop was reduced in the presence of sucrose. The effect was more pronounced for *P. leiognathi* luciferase: fluctuations of its mobile loop at 60 °C in the presence of sucrose became close to the level observed in water at 27 °C.

**Figure 7:**
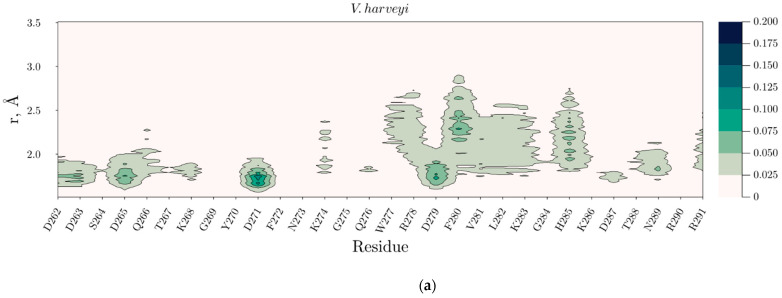

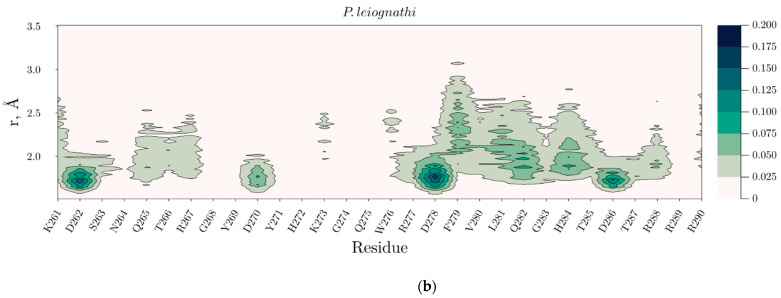
The density map of sucrose molecules near the mobile loop residues of *V. harveyi* (**a**) and *P. leiognathi* (**b**) luciferase at 60 °C during simulation time. The intensity of the green color indicates the probability of sucrose appearance in the distance r from the residue.

The presence of sucrose molecules in the vicinity of amino acids forming the mobile loops of the luciferases during MD modeling was estimated using the minimum-distance distribution function, MDDF [30]. Based on MDDF we constructed the density maps of sucrose appearance at a distance of 1.5–3.5 Å from amino acids of the mobile loops (Figure 7a,b and Figure S6). It turned out that the sucrose atoms located most often within the first solvation shell (in 1.5–2.0 Å) of aspartate negatively charged side chains. Moreover, the density of sucrose molecules near the mobile loop residues of *P. leiognathi* luciferase increases at high temperatures (Figure 7b and Figure S6).

## 3. Discussion

We experimentally compared the temperature effects on two bacterial luciferases in the following directions: (i) the enzymes functioning under various temperatures, (ii) the rates of enzymes’ thermal inactivation, (iii) the thermal denaturation of luciferases. We aimed to reveal signs of protein adaptation to temperature variation for any of two enzymes and to connect them with structural properties of the proteins. However, as thermal adaptation of luminous bacteria could have occurred due to other mechanisms, such as, for example, the accumulation of extremolytes, we tested the influence of sucrose on the temperature dependencies, both functional and structural, of the bacterial luciferases.

The study of reaction kinetics at 5–45 °C revealed the different sensitivity of two luciferases to the temperature of solution (Figure 2 and Figure S1). In particular, *P. leiognathi* luciferase responds by the pronounced activity change for each temperature shift of 5 °C, while *V. harveyi* luciferase provides about the same peak intensity within a wide range of 20–35 °C. The observed thermolability of *P. leiognathi* luciferase and its higher activity under optimum conditions (at 20–25 °C) as compared with *V. harveyi* enzyme are consistent with the conception about the structural and functional features of cold-adapted proteins [31]. Indeed, the activity of *P. leiognathi* luciferase at 10–15 °C, although not maximal, is significantly higher than that of *V. harveyi*. Our molecular dynamics simulations shed light on the structural reasons for this by showing that there are two flexible motifs within the catalytic α-subunit that exhibit distinct mobility in the two luciferases at low temperatures (Figure 6a,b). Apparently, the observed change in structural movements does not provide the necessary mobility of the active site residues of *V. harveyi* enzyme for effective catalysis under cold conditions. Generally, the structure of *V. harveyi* luciferase was more stable at 60 °C as indicated by a smaller standard deviation of RMSD (Table S1). Such behavior is characteristic of proteins capable of functioning at higher temperatures, and not at lower ones. It is worth noting that the majority of known psychrophilic strains of luminous bacteria do belong to the *Photobacterium* and *Aliivibrio* genus, which have “fast” luciferases [15]. Thus, the data on the activity of the studied luciferases at different temperatures are consistent with their phylogeny and molecular dynamics.

Unexpectedly, the empiric kinetic parameter—the decay constant (k_decay_), turned out to be more sensitive to temperature change for the reaction of *V. harveyi* luciferase than of *P. leiognathi* (Figure 2c,d). In the range of 10–30 °C, k_decay_ increases 4.4-fold for the first enzyme and 3.2-fold for the latter. The decay constant is known to depend on rates of three elementary steps of the reaction: the dark decay of peroxyflavin intermediate (k_dd_), aldehyde binding, and the formation of an electronically excited intermediate [32]. The contribution of k_dd_ into k_decay_ substantially differs for two luciferases. In reaction with decanal at 20 °C, the k_decay_ values are 0.32 and 0.21 s^−1^ for the *V. harveyi* and *P. leiognathi* enzymes, whereas the corresponding k_dd_ are 0.06 and 0.15 s^−1^ [33,34]. Thus, distinct temperature dependences of k_decay_ for two luciferases could be caused by two reasons: (i) by a stronger destabilizing effect on peroxyflavin intermediate in *V. harveyi* reaction and/or (ii) by temperature influence on the others than k_dd_ rate constants which could be more pronounced in the *V. harveyi* reaction due to their high contribution into k_decay_. Earlier, we demonstrated that for the reaction of *P. leiognathi* luciferase in the presence of sucrose the rate of the dark decay of peroxyflavin intermediate becomes lower, while the rate of the formation of an electronically excited intermediate increases as compared with the buffer [24]. This could underlie the contrasting changes of k_decay_ observed for two luciferases at 40–45 °C in a sucrose solution (Figure 2c,d).

The possible mechanism of sucrose’s influence on the stability of the reaction intermediates at high temperatures was revealed by molecular dynamics simulations. We observed that at 60 °C the sucrose significantly reduces the fluctuations of the functionally important mobile loop near the active site of *P. leiognathi* (Figure 6d), most likely through direct temporary contacts with aspartate side chains (Figure 7b). For *V. harveyi* luciferase stabilization of the mobile loop by sucrose is less pronounced (Figure 6c), which can cause the increase of the k_decay_ at higher temperatures. Moreover, it was found that sucrose promotes higher fluctuations of αHis150 within the hairpin loop of *V. harveyi* luciferase, which could also influence k_decay_ as well (Figure S4).

Based on the observed temperature effects on the activity of the enzymes in the presence of sucrose, we can conclude that this cosolvent provides no advantages for the functioning of bacterial luciferases either at high (35–45 °C) or at low (5–15 °C) temperatures. Furthermore, we observed that, at low temperatures, sucrose suppresses luciferase activity, probably due to high viscosity which leads to increased diffusion control of the reaction. However, along with activity, the structural stability of proteins also plays an important role in maintenance of their function under extreme conditions, and saccharides are known to be one of the best protein protectors against denaturation [35]. Hence, we further examined the kinetics of thermal inactivation of *V. harveyi* and *P. leiognathi* luciferases in the buffer and in the presence of sucrose. In addition, heat-induced denaturation of the enzymes was investigated using DSC. We observed that under the same conditions the thermal inactivation rate is always smaller for *V. harveyi* luciferase than for *P. leiognathi* (Table 1). Sucrose slows down the thermal inactivation of the enzymes by a factor of two to four times, but without a significant reduction in the activation energy of the process. This sucrose effect could also be a result of a decrease in mobile loop fluctuations at high temperatures, since the enhanced mobility of the protein segments (especially near the active center) could trigger the enzyme transition to an inactive state under heating. The thermal denaturation of the luciferases studied with DSC indicated that the temperature of the maximum of the heat absorption peak (T_m_) is slightly higher for the *V. harveyi* protein than for the *P. leiognathi* one and it increases in sucrose solution. Thus, both approaches showed that *V. harveyi* luciferase has higher temperature stability than *P. leiognathi* and that sucrose is able to protect the proteins against thermal inactivation and denaturation.

In addition, we also found significant differences in ΔH_cal_ between the two luciferases. Many interactions, such as hydration, hydrophobic effect, van der Waals interactions, and hydrogen bonding (between polar surfaces in protein and in water) contribute to the enthalpy of denaturation [36]. Since the spatial structures of *P. leiognathi* and *V. harveyi* luciferases are very similar, the difference in ΔH_cal_ can be explained by the presence of a slightly larger number of unstructured parts in *V. harveyi* luciferase compared to *P. leiognathi* luciferase. This was confirmed by our early data on CD spectroscopy: *V. harveyi* luciferase has slightly less secondary structure compared to *P. leiognathi* luciferase [37]. According to our MD data, we also see that the hairpin loop within the *V. harveyi* structure was more flexible than in the *P. leiognathi* structure at high temperatures. It is worthy of note that our previous investigation of the unfolding of *V. harveyi* and *P. leiognathi* luciferases with urea showed that the latter has a more stable tertiary and secondary structure [37]. In agreement with these results, our current data on the enthalpy of thermal denaturation also indicate that *P. leiognathi* luciferase has a greater number of intramolecular interactions, which are disrupted with increasing temperature (parameter ΔH_cal_ in Table 2).

The chemical unfolding of the bacterial luciferase can be considered much more studied than thermal denaturation [38]. A number of investigations have proven a four-state scheme for denaturation of *V. harveyi* luciferase with urea [39,40], where the unfolding of the C-terminal domain of the α-subunit is followed by the dissociation of the two subunits and the subsequent denaturation of them. In our previous work [37], we demonstrated that *P. leiognathi* luciferase in urea solutions follows the same unfolding pathway. However, the results presented in this work suggest that the mechanism of thermal denaturation of the two studied enzymes could differ. This is not very surprising, since chemical and thermal denaturation processes do not have to correlate, as the denaturant interacts specifically with the protein, while temperature generally affects intramolecular contacts indiscriminately [41].

Lack of knowledge about thermostability and the activity of bacterial luciferase at different temperatures is one of the issues when it is implemented as a reporter for the real-time monitoring of various processes in microbial or eukaryotic cells [42,43,44]. Previous studies demonstrated that in order to obtain a reproducible luminescent response and to understand this response, a detailed analysis of the characteristics of the proteins themselves is necessary, as well as the influence of the environment on their structure and function [43,45]. Therefore, our data has significant implications for the use of two types of luciferase with distinct sensitivity to temperature in terms of both activity and stability as reporters.

## 4. Materials and Methods

### 4.1. Materials

Lyophilized recombinant luciferase *P. leiognathi* (99% purity) was purchased from Biolumdiagnostika Ltd. (Krasnoyarsk, Russia). Luciferase *V. harveyi* was lyophilized after expression in the *E. coli* strain BL21 (DE3), and purification was as described in [37] in the Photobiology laboratory of the Institute of Biophysics SB RAS (Krasnoyarsk, Russia). Flavin mononucleotide (FMN, Sigma-Aldrich, Burlington, MA, USA) and decanal (Acros Organics, Fair Lawn, NJ, USA) were used as luciferase substrates. Ethylenediaminetetraacetic acid (EDTA, ROTH) was applied as an electron donor during FMN photoreduction. Sucrose (Gerbu, Heidelberg, Germany) in a concentration of 30 wt% was used as a model extermolyte. Stock solutions of the reactants were prepared in a potassium phosphate buffer (0.05 M, pH 6.9), except for decanal, which was dissolved in ethanol (2 × 10^−3^ M). The concentrations of FMN and luciferase were determined spectrophotometrically with the extinction coefficients of ε_445_ = 12,400 M^−1^ cm^−1^ and ε_280_ = 80,000 M^−1^ cm^−1^, respectively.

### 4.2. Activity and Thermal Inactivation of Bacterial Luciferases

The reaction kinetics for the luciferases was measured at 5–45 °C in a single-turnover assay using a SX20 stopped-flow spectrometer (Applied Photophysics, Leatherhead, UK) as described elsewhere [24]. Decanal and photoreduced flavin mononucleotide in the buffer or in the sucrose solution were loaded into one drive syringe of the spectrometer and pre-incubated there for 5 min. Similarly, bacterial luciferase in the buffer or sucrose solution was pre-incubated for 5 min in another drive syringe. The enzyme and substrates were then mixed in the measuring cell and the kinetics of light emission was registered for 10–30 s. The cell and drive syringes were thermostated at the required temperature. The concentrations in the reaction mixtures were 0.5 µM for luciferase, 15 µM for flavin and 50 µM for decanal.

The thermal inactivation rate of the luciferases was estimated by measuring the remaining activity of the enzymes after their incubation for various times under the required temperature in the range 40–55 °C. Solid-state thermostat Gnom (DNA technology, Moscow, Russia) was used for enzyme incubation. Reaction kinetics were measured at 20 °C in a single-turnover assay with a SX20 stopped-flow spectrometer as described above, but without incubation in drive syringes. The remaining activity (R) of luciferase was then calculated by percentage according to R = (Q*/Q_0_*)·100, where Q* and Q_0_* are areas under the kinetic curves obtained with thermally treated and thermally untreated enzymes, respectively. The rate constant of thermal inactivation, *k*, was determined by fitting the dependence of R on incubation time, t, with the function R = A∙*e*^−*k*t^ using Origin 8.0 software (OriginLab, Northampton, MA, USA). The coefficient of determination, R^2^, was >0.96 for all of the approximations. The activation energy of thermal inactivation (E_a_) was calculated according to the Arrhenius equation from the slope of dependence ln*k* (1/T).

### 4.3. Differential Scanning Calorimetry

Calorimetric measurements were made using a precision scanning microcalorimeter SCAL-1 (Scal Co. Ltd., Pushchino, Russia) with 0.33-mL glass cells at a scanning rate of 1 K/min and under 2.5 atm pressure [46]. The protein concentrations ranged from 1.0 to 4.0 mg/mL in 50 mM potassium-sodium phosphate buffer with a pH of 6.9. In this concentration range, the thermodynamic parameters remained stable. The experimental calorimetric profiles were corrected for the calorimetric baseline, and the molar partial heat capacity functions were calculated in a standard manner. The excess heat capacity, C_p_^exp^, was evaluated by subtracting the linearly extrapolated initial and final heat capacity functions with correction for the difference of these functions by using a sigmoid baseline [47]. A typical value for the partial specific volume for globular proteins (0.73 cm^3^/g) was accepted arbitrarily, since it does not influence the calculated excess heat capacity. In the calculation of molar thermodynamic quantities, the molecular weight used was 80,000 Da, which corresponds to the dimer state of the luciferases.

### 4.4. MD Simulation

The previously prepared structures of the *V. harveyi* and *P. leiognathi* luciferases [37] were taken for molecular dynamics simulations (MD) at different temperatures (5, 15, 27, 45, 60 °C) using the GROMACS 2020.4 software package and CHARMM36 force field [48,49]. The enzymes were solvated in a cubic box with periodic boundary conditions and at least 12 Å away from the protein to each of the box edges. Two strategies were applied for the solvation procedure: (1) TIP3P explicit water [50] was used for simulation at all studied temperatures; and (2) a mixture with sucrose molecules [51] adjusted to simulate the concentration at 30 wt% was exploited for molecular dynamics at 300 and 333 K. The net charge of the systems was neutralized with sodium ions. The minimization of each system was performed with the steepest descent method (maximum force of 1000.0 kJ/mol). The system was then heated, and sequential execution of isochoric and isobaric equilibrations was performed for 10 ns each with a 2 fs time step. A V-rescale thermostat at the required temperature and a Parrinello–Rahman barostat at 1 bar of pressure was used. Protein heavy atoms were restrained during equilibration [52,53]. The cutoff distance for the short-range non-bonded interactions was 10 Å. Electrostatic interactions were treated using the particle-mesh Ewald method [54]. An integration step of 2.0 fs was used, and bonds were constrained with the LINCS algorithm [55]. We conducted 100-ns molecular dynamics simulations for each system. Each MD run was repeated three times to obtain the average value of the structural parameters for the studied proteins.

## 5. Conclusions

In this study we aimed at comparing the effect of different temperatures on the structure and function of two bacterial luciferases: the *Vibrio harveyi* and *Photobacterium leiognathi* species. Despite high homology, these enzymes belong to different subfamilies with specific structural features of the active center and distinct reaction kinetics [20]. Thus, *V. harveyi* luciferase is classified as a “slow” enzyme, whereas *P. leiognathi* luciferase is a “fast” one. To the best of our knowledge, systematic studies of the thermolability of “slow” and “fast” bacterial luciferases has yet to be carried out.

The results of our study indicate that the “fast” *P. leiognathi* luciferase is thermolabile and has maximal activity in a narrower temperature range than does the “slow” *V. harveyi* luciferase. The latter is weakly sensitive to temperature change and remains active up to 35 °C. However, *P. leiognathi* luciferase provides twice the total quantum yield per single turnover under optimal conditions. At lower temperatures, the activity of the “fast” enzyme is significantly higher compared to the “slow” one as well. This is probably due to the peculiarities of its functional mobile loop dynamics, resulting in the ability to more effectively stabilize the reaction intermediates reflected in the smaller decay constant of the reaction. Moreover, our data revealed an interesting pattern that despite the greater number of intramolecular contacts disrupted during the unfolding of *P. leognathi* luciferase, it has slightly lower temperature stability. Thus, luciferase with the fast decay kinetics demonstrates the properties of the cold-adapted enzymes, while luciferase with the slow kinetics is more resistant to high temperatures. All of this reflects the well-known interplay between the activity and structural stability of enzymes, implying that more stable proteins could not provide a high rate of catalysis due to the rigidity of the structure.

In addition, we studied the effect of sucrose on the stability and activity of two bacterial luciferases. Our investigation has revealed that sucrose does not stabilize both enzymes in terms of activity, but it can protect the proteins from thermal denaturation. It is noteworthy that the influence of sucrose on the denaturation mechanisms of two luciferases seems to be different and requires more detailed examination. 

Thus, after experimental research and molecular dynamics modeling of temperature effects. we have found that, despite high homology, the *V. harveyi* and *P. leiognathi* luciferases exhibit distinct features in response to heating. However, to answer the question of whether the found structural and functional peculiarities are a sign of the entire bacterial luciferase subfamily (“slow” or “fast”) or is an individual feature of only the studied enzymes, further research is required.

## Figures and Tables

**Table 1:** The thermal inactivation rate constants for the *P. leiognathi* and *V. harveyi* luciferases in the buffer and sucrose solutions (30%) at different temperatures.

*V. harveyi*			*P. leiognathi*		
T, °C	k, min^−1^		T, °C	k, min^−1^	
	Buffer	Sucrose		Buffer	Sucrose
45	0.15 ± 0.01	0.07 ± 0.01	40	0.02 ± 0.001	0.01 ± 0.001
48	0.71 ± 0.11	0.17 ± 0.01			
50	1.36 ± 0.10	0.39 ± 0.02	45	0.12 ± 0.01	0.05 ± 0.01
52	1.63 ± 0.24	0.80 ± 0.06	50	0.52 ± 0.01	0.15 ± 0.01
55	2.69 ± 0.32	1.14 ± 0.05	55	1.10 ± 0.03	0.56 ± 0.05

**Table 2:** The thermodynamic parameters of the thermal denaturation of the bacterial luciferases *.

Luciferase	Solvent	T_m_, ±0.1 K (°C)	ΔT_1/2_, ±0.1 K	ΔH_cal_, kJ/(K⋅mol)	ΔQ_−_/ΔQ_+_
*P. leiognathi*	Buffer	318.5 (45.4)	4.2	997 ± 80	1.3
	30% sucrose	323.2 (50.1)	6.3	850 ± 50	1.6
*V. harveyi*	Buffer	320.1 (47.0)	7.4	552 ± 45	1.3
	30% sucrose	325.1 (52.0)	7.5	252 ± 30	1.3

* T_m_—the temperature of the maximum of heat absorption peak; ΔT_1/2_—half-width of the heat absorption peak; ΔH_cal_—calorimetric enthalpy; ΔQ_−_/ΔQ_+_—degree of asymmetry, where ΔQ_−_—heat released when warming up to temperature T_m_, ΔQ_+_—heat released after T_m_.

## Data Availability

The data presented in this study are available on request from the corresponding author.

## Supplementary Materials

The following supporting information can be downloaded at: https://www.mdpi.com/article/10.3390/ijms23158119/s1.
