# Supplementary material for: Structure-Function Relationships in Temperature Effects on Bacterial Luciferases: Nothing Is Perfect
Source: Int J Mol Sci. 2022 Jul 23;23(15):8119. doi: 10.3390/ijms23158119 (PMC9332260; doi:10.3390/ijms23158119)
Supplement: Supplementary file 1 [file ijms-23-08119-s001.zip › ijms-1829179-supplementary.pdf]

Structure-Function Relationships in Temperature Effects on Bacterial Luciferases: Nothing Is Perfect

Anna A. Deeva [1], Albert E. Lisitsa [1], Lev A. Sukovatyi [1], Tatiana N. Melnik [2], Valentina A. Kratasyuk[1,3], and Elena V. Nemtseva[1,3]

[1] Biophysics Department, Siberian Federal University, Krasnoyarsk, Russia

[2] Institute of Protein Research, Russian Academy of Sciences, Pushchino, Moscow Region, Russia

[3] Institute of Biophysics, Siberian Branch of Russian Academy of Sciences, Krasnoyarsk, Russia

Supplementary Material

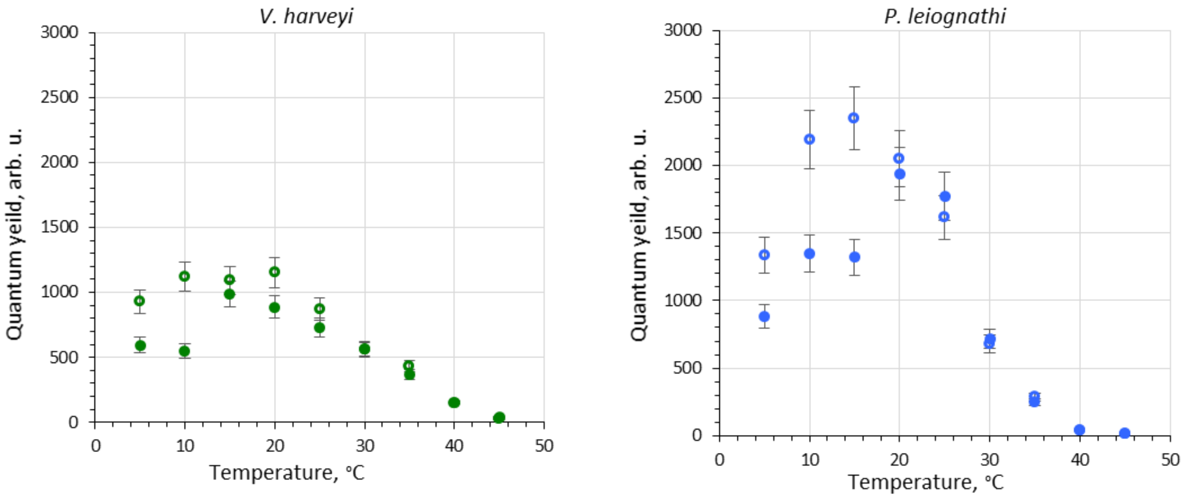

(**a**) (**b**)

Figure S1. The dependence of total quantum yield of a single turnover, Q*, of luciferases from *V. h rveyi* (a) and *P. leiognathi* (**b**) on temperature in the buffer (empty circles) and sucrose solution (filled circles).

Table S1. Structural parameters of *V. harveyi* and *P. leiognathi* luciferases at different temperatures: the root-mean-square deviation of the backbone atoms (RMSD), the radius of gyration (R_g), and the solvent accessible surface area (SASA). Each parameter was calculated for the last 10 ns of three MD-trajectories. Data presented are the average ± standard deviation.

Parameter	Type of luciferase	5 °C	15 °C	27 °C		45 °C	60 °C	
		Water	Water	Water	30% Sucrose	Water	Water	30% Sucrose
RMSD, Å	*V. harveyi*	1.7±0.2	1.8±0.1	1.7±0.1	1.6±0.2	2.2±0.1	2.3±0.3	2.1±0.3
	P. leiognathi	2.2±0.2	2.2±0.2	2.2±0.2	1.9±0.2	2.6±0.2	2.6±0.5	2.4±0.2
Rg., Å	*V. harveyi*	26.6±0,1	26.6±0,1	26.7±0.1	26.7±0.1	26.8±0.1	26.8±0.1	26.7±0.1
	P. leiognathi	27.0±0.1	27.1±0.2	26.8±0.1	27.0±0.1	27.1±0.1	27.1±0.2	27.2±0.2
SASA × 10^2, Å^2	*V. harveyi*	287±5	284±4	286±4	287±4	288±5	290±7	286±4
	P. leiognathi	298±4	300±12	293±5	294±3	295±6	297±7	301±8

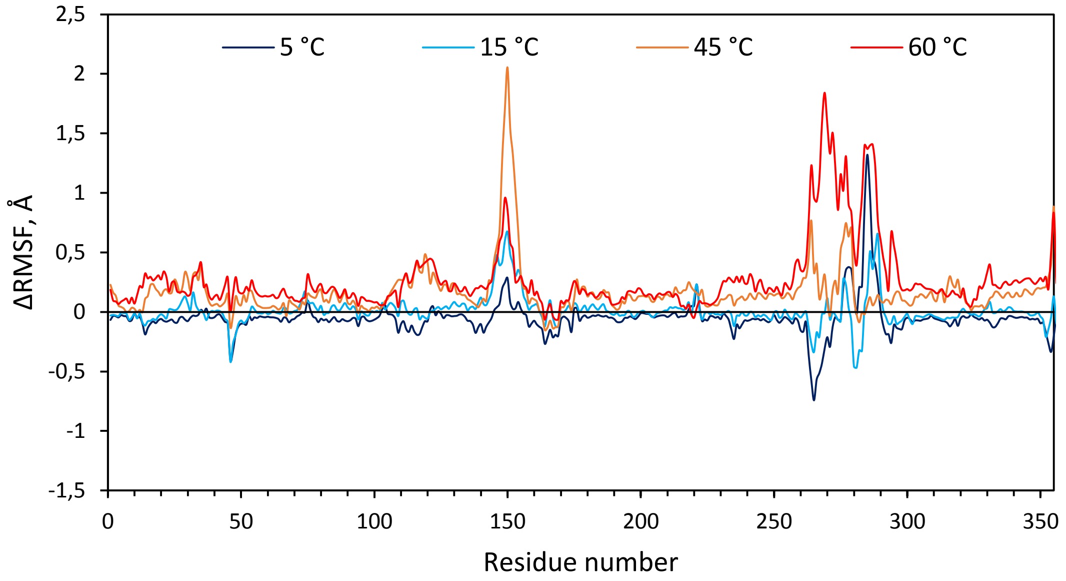

(**a**)

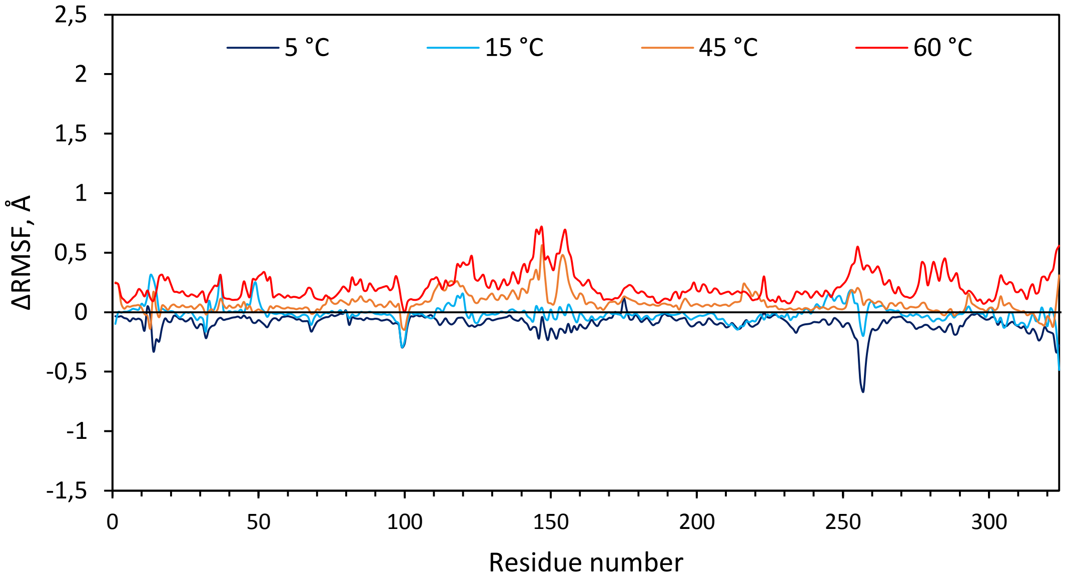

(**b**)

Figure S2. ΔRMSF of C$_{\alpha}$ atoms in the *α*-subunit (**a**) and the β-subunit (**b**) of *V. harveyi* luciferase in water at different temperatures. The positive value of ΔRMSF corresponds to a more flexible segment, as compared with the structure in water at 27 °C, while the negative ΔRMSF corresponds to a more rigid segment.

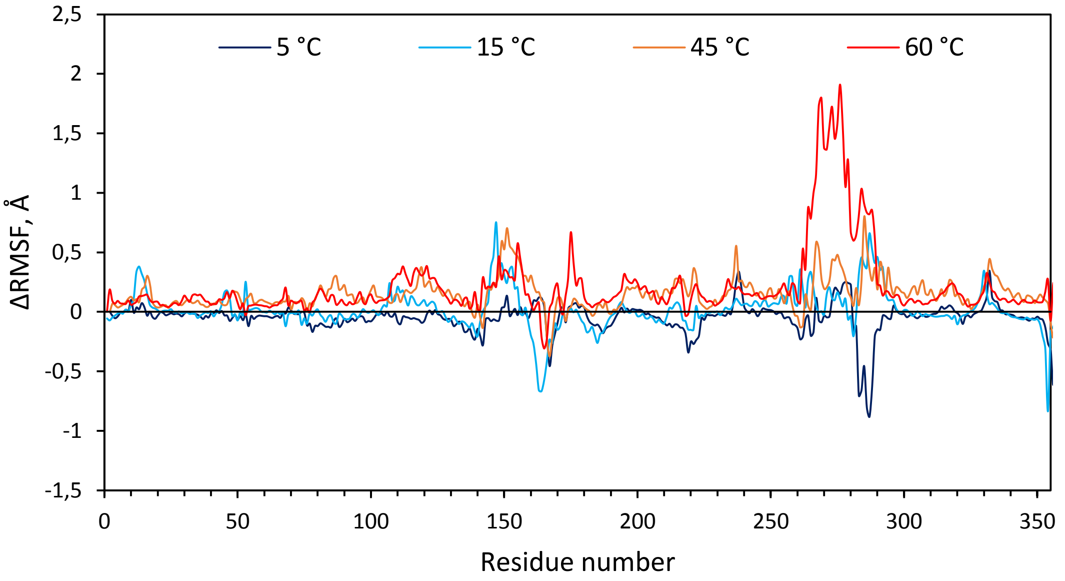

(**a**)

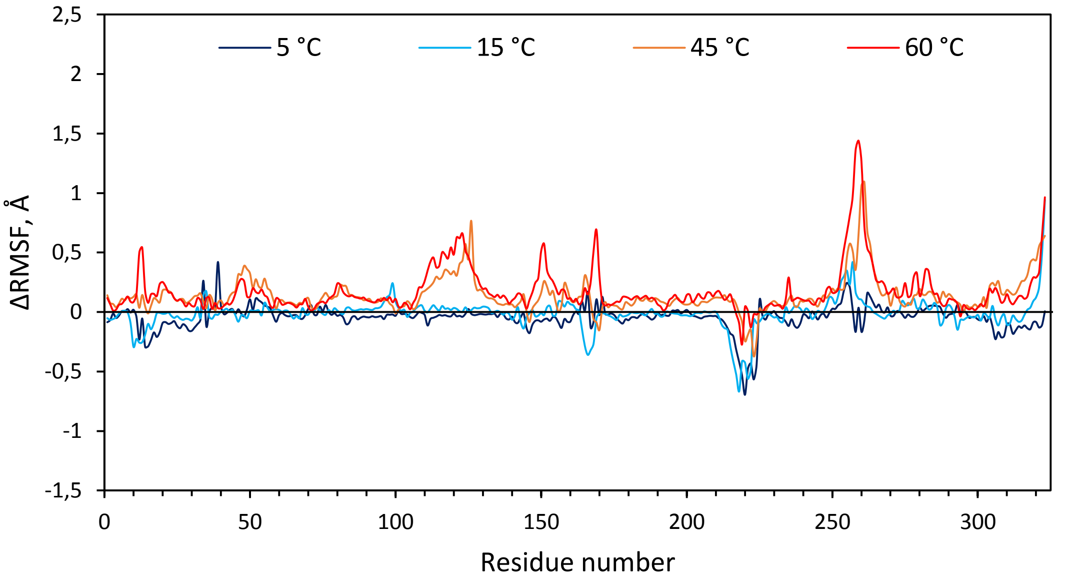

(**b**)

Figure S3. ΔRMSF of C_α atoms in the *α*-subunit (**a**) and the β-subunit (**b**) of *P. leiognathi* luciferase in water at different temperatures. The positive value of ΔRMSF corresponds to a more flexible segment, as compared with the structure in water at 27 °C, while the negative ΔRMSF corresponds to a more rigid segment.

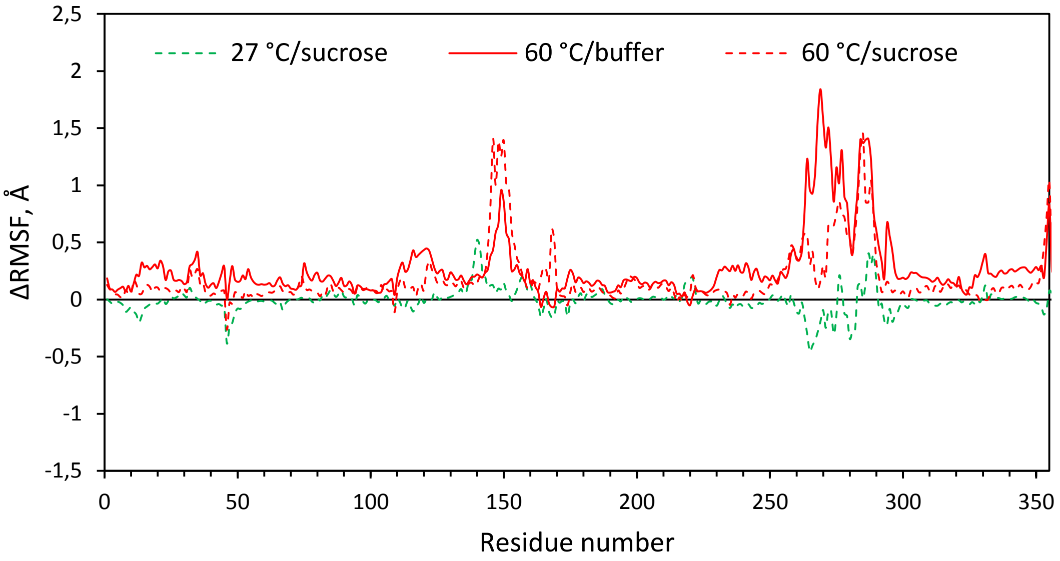

(**a**)

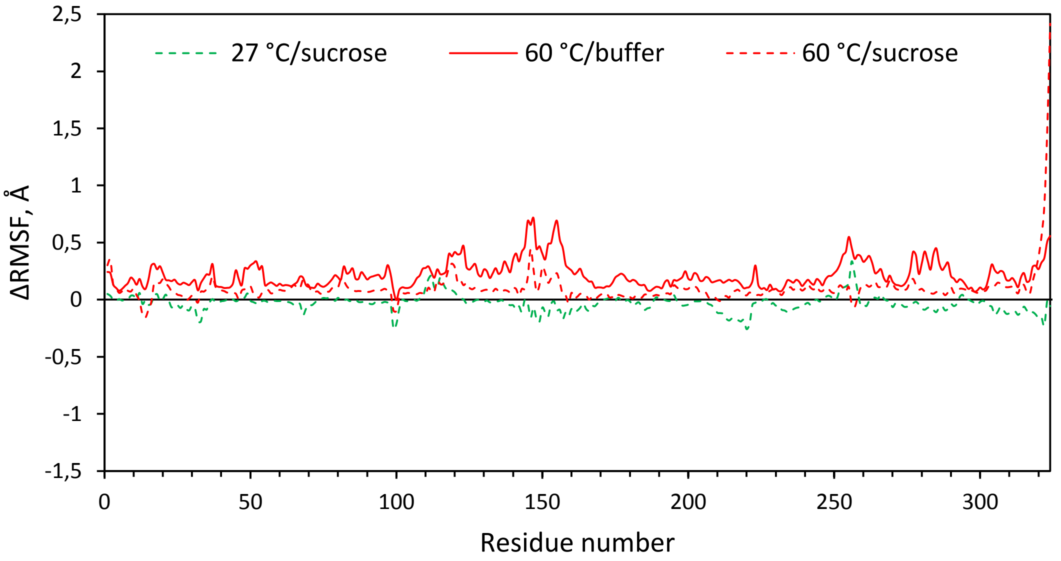

(**b**)

Figure S4. ΔRMSF of C_α atoms in α-subunit (**a**) and β-subunit (**b**) of *V. harveyi* luciferase in water-sucrose (30%) at different temperatures. The positive value of ΔRMSF corresponds to a more flexible segment, as compared with the structure in water at 27 °C, while the negative ΔRMSF corresponds to a more rigid segment.

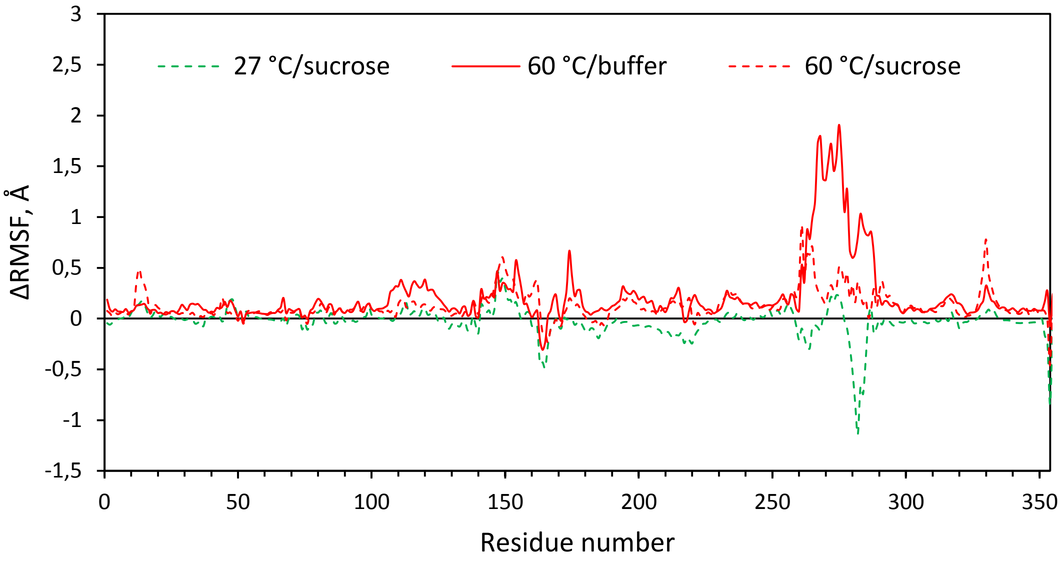

(**a**)

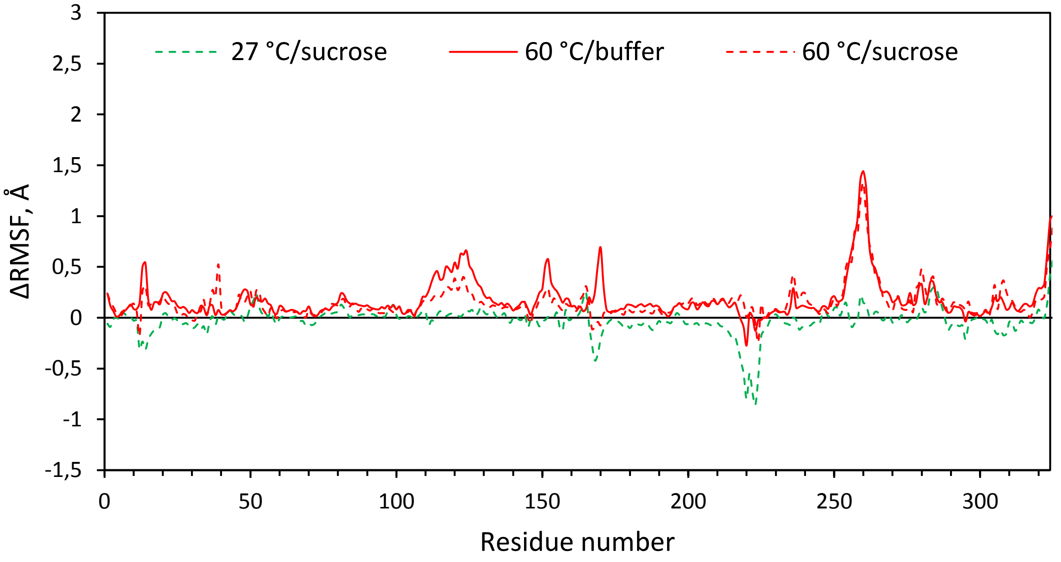

(**b**)

Figure S5. ΔRMSF of C_α atoms in *α*-subunit (**a**) and β-subunit (**b**) of *P. leiognathi* luciferase in water-sucrose (30%) at different temperatures. The positive value of ΔRMSF corresponds to a more flexible segment, as compared with the structure in water at 27 °C, while the negative ΔRMSF corresponds to a more rigid segment.

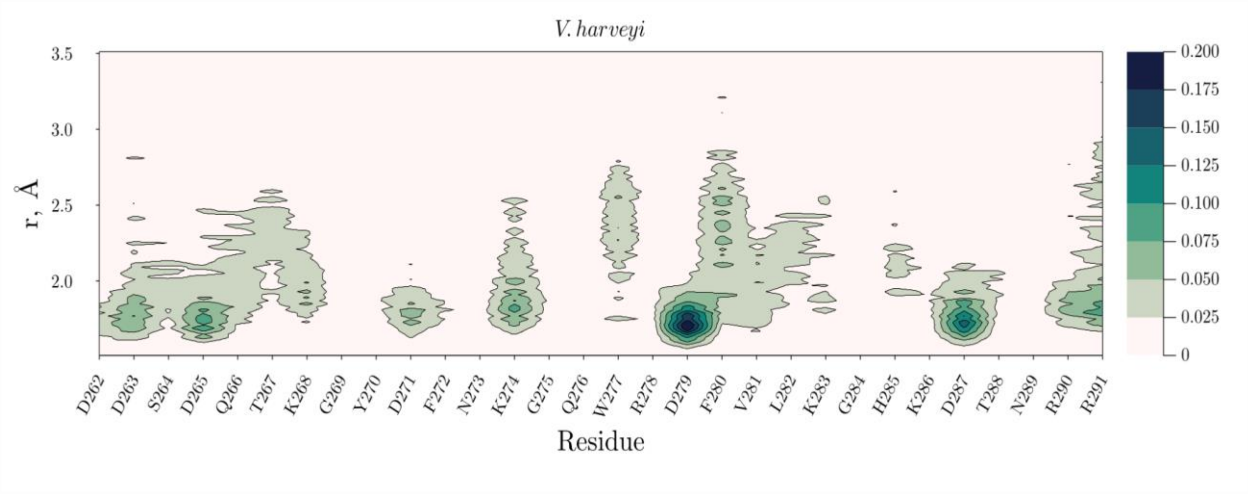

(**a**)

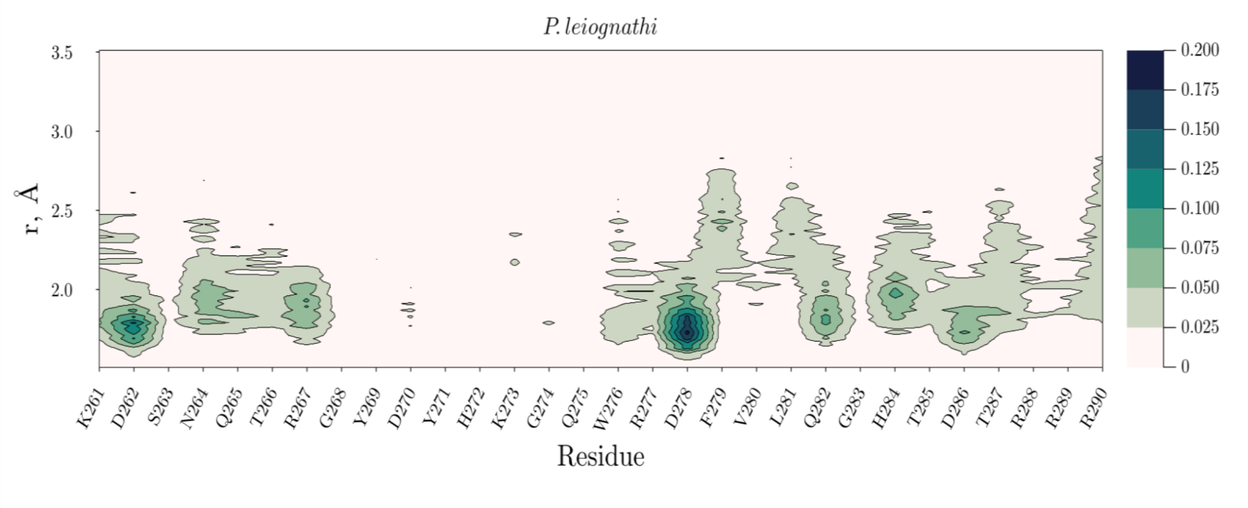

(**b**)

Figure S6. The density map of sucrose molecules near the mobile loop residues of *V. harveyi* (**a**) and *P. leiognathi* (**b**) luciferases at 27 °C during simulation time. The intensity of the green color indicates the probability of sucrose appearance in the distance ***r*** from the residue.

Table S2. Correlation Coefficients between ΔRMSF profiles of *V. harveyi* and *P. leiognathi* mobile loops at different temperatures.

5 °C	15 °C	45 °C	60 °C	27 °C/sucrose	60 °C/sucrose
-0,52	0,69	-0,27	0,67	0,12	-0,22
